# Feasibility of “DiverAcción”: A Web-Based Telerehabilitation System for Executive Functions Training in Children and Adolescents with ADHD—Longitudinal Study Protocol

**DOI:** 10.3390/healthcare14030323

**Published:** 2026-01-27

**Authors:** Marina Rivas-García, Carmen Vidal-Ramírez, Abel Toledano-González, María del Carmen Rodríguez-Martínez, Esther Molina-Torres, José-Antonio Marín-Marín, José-Matías Triviño-Juárez, Miguel Gea-Mejías, Dulce Romero-Ayuso

**Affiliations:** 1 Department of Physical Therapy, Division of Occupational Therapy, University of Granada, 18006 Granada, Spain; mrgmarina@correo.ugr.es; 2 Department of Radiology and Physical Medicine, University of Granada, 18016 Granada, Spain; carmenvidal@correo.ugr.es (C.V.-R.); jmtjuarez@ugr.es (J.-M.T.-J.); 3 Department of Physical Therapy, University of Málaga, 29071 Málaga, Spainmarrodmar@uma.es (M.d.C.R.-M.); 4 Department of Didactics and School Organzation, University of Granada, 18071 Granada, Spain; esthermolinatorres@gmail.com (E.M.-T.);; 5 Department of Software Engineering, University of Granada, 18071 Granada, Spain; mgea@ugr.es; 6 Brain, Mind and Behaviour Research Center (CIMCYC), University of Granada, 18071 Granada, Spain; 7 Instituto de Investigación Biosanitaria ibs.Granada, 18014 Granada, Spain

**Keywords:** ADHD, executive functions, stress, telerehabilitation, digital health, feasibility study, prospective memory, planning skills, children, adolescents, ecological validity

## Abstract

**Background**: Attention Deficit Hyperactivity Disorder (ADHD) is associated with executive function deficits—such as planning, organization, and prospective memory—that impair autonomy and daily functioning, increase family stress, and create challenges in educational contexts. These consequences underscore the need for accessible and ecologically valid interventions addressing the cognitive, familial, and educational dimensions. Traditional approaches often lack ecological validity, and pharmacological treatment shows a limited impact on functional cognition. **Objectives**: This protocol outlines a feasibility study of DiverAcción, a web-based telerehabilitation system designed to enhance functional cognition through interactive and gamified tasks integrated into a comprehensive healthcare programme. **Methods**: A quasi-experimental feasibility study before and after the study will recruit 30 participants aged 9 to 17 years with ADHD. The study comprises an initial face-to-face session for instructions and baseline assessment (T0), followed by twelve supervised online sessions over six weeks. Therapeutic support is provided via integrated chat, email, and two scheduled videoconference check-ins. **Feasibility Outcomes**: include recruitment, adherence, retention, usability (SUS), acceptability (TAM), satisfaction, user-friendly design, therapeutic alliance (WAI-I), and professionals’ attitudes toward technology (e-TAP-T). **Exploratory Measures**: include parental self-efficacy (BPSES), parenting stress (PSI-4-SF), ADHD symptomatology (SNAP-IV), executive functioning (BRIEF-2), time management (Time-S), emotional regulation (ERQ-CA), prospective memory (PRMQ-C), and health-related quality of life (KIDSCREEN-52). Analyses emphasize descriptive statistics for feasibility metrics (recruitment, adherence, retention, dropout and fidelity). Assessments are conducted post-intervention (T1) and at three-month follow-up (T2) and analyzed relative to baseline using repeated-measures ANOVA or Friedman tests, depending on data distribution. **Conclusions:** This feasibility protocol will provide preliminary evidence on the usability, acceptability, and implementation of DiverAcción. Findings will guide refinements and inform the design of a subsequent randomized controlled trial.

## 1. Background

Attention-Deficit/Hyperactivity Disorder (ADHD) is one of the most common neurodevelopmental conditions in childhood and adolescence (≈5–7%), and is associated with executive function (EF) deficits—particularly planning, organization, and prospective memory (PM)—that compromise autonomy in daily living, school participation, and family well-being. These characteristics highlight the need for interventions with strong ecological validity that address not only cognitive processes but also the family and educational contexts where change must occur. Pharmacological treatment remains first-line, yet its impact on functional cognition is limited; likewise, many psychological approaches show modest transfer to real-world performance [1,2].

Across development, EF follows a non-linear trajectory with rapid gains from early to mid-adolescence, decelerating improvement toward late adolescence and relative stabilization into adulthood; complex planning may continue to improve into the early twenties. PM also shows distinct profiles for event-based versus time-based demands, with the latter placing heavier self-monitoring loads. In ADHD, EF and PM often present uneven and protracted trajectories alongside common difficulties in emotion regulation (ER), which can exacerbate task initiation and execution. These findings have direct implications for intervention design: scaffolding metacognition (plan–monitor–evaluate), grading autonomy by age band, and explicitly supporting time-based PM and ER are key to feasibility and transfer [3,4,5,6,7,8,9,10,11].

Traditional cognitive rehabilitation has prioritized isolated process training (e.g., attention, memory) using repetitive, decontextualized tasks. Evidence suggests that improvements achieved in clinic rarely generalize to meaningful participation. In contrast, functional cognition refers to how cognitive abilities underlying occupational performance are integrated and applied to plan, organize, monitor, and complete meaningful tasks in real-world contexts, thus supporting autonomy and occupational participation. This paradigm emphasizes strategy use within everyday activities, environmental adaptations, and personally relevant goals to enhance transfer and sustained change [12,13,14,15].

Occupational therapy programs for ADHD adopt ecological approaches combining explicit EF training with caregiver mediation. Cog-Fun demonstrates short-term improvements by training planning, organization, inhibition, monitoring, and ER through meaningful, family-involved activities (≈10 sessions in childhood; 17–20 in adolescence). POET provides eight sessions of parent-focused coaching (home routines, parental EF), improving routine management and perceived competence; POET-T adapts the model to teachers and classroom cycles (6–8 weeks), enhancing participation though with limited global EF change. These models share ecological validity but differ in agents, dosage, and delivery context, and there remains a gap regarding multimodal integration and explicit training of time-based PM to support cross-context generalization [16,17,18,19,20,21,22].

Emerging digital tools—including virtual reality and web-based telerehabilitation—enable controlled yet realistic task demands, facilitate motivation and accessibility, and can reduce logistical barriers while supporting strategy transfer to natural contexts. DiverAcción was conceived to address the above gaps through an integrated, ecologically valid, web-based program that (1) explicitly trains EF (planning, organization, time management) and PM (with emphasis on time-based demands), (2) embeds ER supports, and (3) actively involves parents and teachers to promote generalization across home–school settings. The program combines a collaborative agenda (daily plan–monitor–evaluate scaffold), gamified training sessions, and structured caregiver sessions/supervision, aligning developmental needs with age-banded adaptations [23,24].

Finally, consistent with international guidance for developing child–adolescent digital mental health interventions and with the SPIRIT/CONSORT-eHealth framework, a feasibility evaluation is a necessary first step prior to an efficacy randomized controlled trial, focusing on usability, acceptability, adherence, and preliminary change signals in functional cognition domains relevant to everyday life [25,26,27,28].

## 2. Methods

### 2.1. Design

This feasibility study of DiverAcción adopts a quasi-experimental pretest-posttest design without a control group, following the Medical Research Council (MRC) guidelines for complex interventions [29,30] and the SPIRIT 2025 guidelines [26]. This protocol was developed and reported in accordance with the CONSORT extension for pilot and feasibility trials, the SPIRIT 2025 guidelines for trial protocols, and the CONSORT-eHealth checklist for digital interventions [26,27,28]. This is a feasibility trial; a full protocol registration will be completed after analyzing feasibility outcomes and incorporating any necessary adaptations. The trial registry identifier will be provided once the definitive protocol is submitted. The Supplementary Material provides an integrated checklist for each guideline.

### 2.2. Study Setting

The study will be conducted by the local university, in collaboration with local hospitals, health centers, the child and adolescent mental health unit, and the educational guidance team specializing in severe behavioral disorders. The intervention sessions will be conducted entirely online through the DiverAcción website, accessible via computer or tablet, and supervised by therapists from the research team.

### 2.3. Participants and Recruitment

Purposive sampling will be used to ensure the representativeness of different profiles of children and adolescents with ADHD. Participants and their primary caregivers will be recruited through the local Specialized Educational Guidance Team for Severe Behavioral Disorders, which will distribute invitations through school guidance counselors. Additionally, neuropediatricians from local hospitals and health centers will collaborate.

Potential candidates who meet the criteria and express interest in participating will be informed. A researcher from the team will contact eligible participants by telephone to confirm their inclusion and explain the objectives and characteristics of the study.

#### 2.3.1. Inclusion Criteria

- Age between 9 and 17 years.
- Functional proficiency in Spanish (oral and written).
- Primary educational or clinical diagnosis of ADHD.
- Intelligence quotient (IQ > 69). Intellectual functioning will be assessed using the Wechsler Abbreviated Scale of Intelligence—Second Edition (WASI-II) [31]. The WASI-II is a standardized abbreviated intelligence test designed for individuals aged 6 to 90 years. It provides an estimate of Full-Scale IQ (FSIQ) based on two subtests (Vocabulary and Block Design) or four subtests (Vocabulary, Similarities, Block Design, and Matrix Reasoning). The WASI-II demonstrates high reliability (α ≈ 0.90–0.95) and strong validity, with correlations exceeding r = 0.90 with the full WAIS-IV and WISC-V scales, making it suitable for research purposes where a brief yet accurate measure of intellectual functioning is required [32].
- T-score ≥ 65 on the BRIEF-2 Cognitive Regulation and Behavioral Regulation indices, reported by parents or teachers.
- Informed consent from the legal guardian and written assent from the minor.

#### 2.3.2. Exclusion Criteria

- Sensory or motor limitations that prevent the use of electronic devices (computer, tablet, or mobile phone) reported by caregivers or pediatricians
- Photosensitive epilepsy, reported by pediatricians.
- Uncontrolled aggressive behavior, reported by local Specialized Educational Guidance Team for Severe Behavioral Disorders or pediatricians

### 2.4. Intervention

#### 2.4.1. Overview and Theoretical Basis

**Figure 1:**
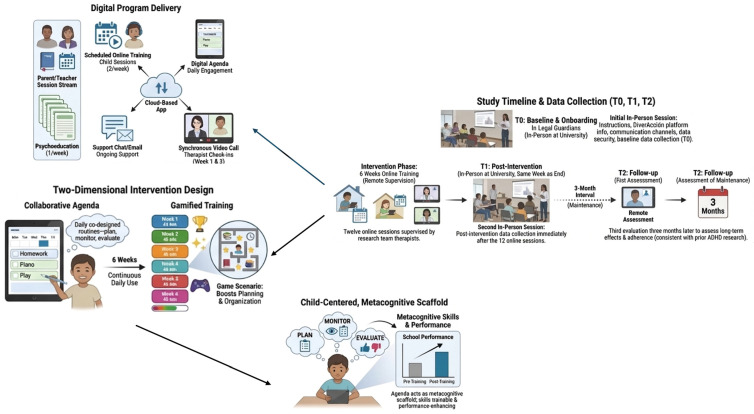
DiverAcción Intervention and timeline of the different phases of the study.

DiverAcción is a digital telerehabilitation system designed to train executive skills—planning, organization, and time management—and emotional self-regulation in virtual contexts with high ecological validity (Table 1; Figure 1). The program is grounded in organizational skills training for children with ADHD, adapted to interactive digital environments [26,33], and in Adele Diamond’s model of executive functions [6].

The intervention emphasizes planning and organization, acknowledging a non-linear EF trajectory with rapid gains at 10–15, decelerating improvement at 15–18, and stabilization toward 18–20 [3]; complex planning can continue improving into the late teens/early twenties [4]. Planning matures later than working memory and inhibition [5]. The design also incorporates the development of prospective memory (PM)—event-based vs. time-based demands—and its reliance on executive control [8,9,34].

#### 2.4.2. Components of the Intervention

The intervention comprises two core dimensions: (1) Collaborative agenda, and (2) Training. The collaborative scheduling component is co-designed with participants to improve and apply planning, organizational, and time management skills in daily life. It is intended for daily use to establish a routine of planning and monitoring activity completion. In parallel, each participant will complete twelve 45-min virtual training sessions, delivered twice per week across six weeks. Training activities are gamified to enhance motivation while targeting planning, organization, time management, and emotional self-regulation.

#### 2.4.3. Child-Centered, Metacognition-Supportive Agenda

The program will be child-centered: each participant will construct a personal agenda that aligns training tasks with daily routines and demands. The agenda will function as a metacognitive scaffold (plan–monitor–evaluate), nurturing planning and self-regulation [35,36]. Evidence indicates that metacognitive skills are trainable in school settings and uniquely contribute to performance beyond intellectual ability [37,38].

#### 2.4.4. Delivery Format and Dosage

The program will be delivered entirely in a digital, remote format over six weeks. Participants will engage in scheduled online training activities, utilize the digital agenda for planning and organization, and attend synchronous videoconference sessions led by trained occupational therapists. The six-week protocol comprises 12 child/adolescent sessions (two per week) and six parent/teacher sessions (one per week), aligning with the dosage planned for the subsequent randomized controlled trial. In addition to scheduled sessions, participants will be instructed to use the collaborative agenda daily (seven days per week) throughout the intervention to support continuous engagement and transfer of strategies to natural home–school contexts.

#### 2.4.5. Developmental Adaptations (EF, ER, PM)

Implementation was scaled to age-related EF capacities and emotion-regulation needs, grounded in contemporary neurodevelopmental models [6] and adolescent emotion circuitry [7]. his age-adjustment approach is consistent with prior interventions targeting planning and organization, which tailor content with age-appropriate task sets (e.g., one set for 6–9 and another for 10–13 years) [39]. In our study, participants will be grouped into three bands (9–11, 12–14, 15–17) to align task demands with developmental stages of EF, ER, and PM [9,34]: (a) 9–11 years: Greater reliance on external scaffolds, salient event cues, and guided time monitoring; event-based PM benefits from cues, whereas time-based PM requires more self-initiated monitoring [8,11]; (b) 12–14 years: Increased emotional reactivity with emerging, inconsistent self-initiated control; tasks emphasized structured monitoring, graded autonomy, and explicit ER supports [7,40]; (c) 15–17 years: More advanced planning and prospective organization, but vulnerability under high cognitive/emotional load and complex PM paradigms; encouraged self-monitoring, anticipatory planning, cognitive reappraisal, and autonomous compensatory tools (e.g., digital reminders for time-based PM) [34,41]. In ADHD, PM shows uneven trajectories and deficits—often more pronounced for time-based PM due to higher monitoring demands—and ER difficulties are common; strengthening working memory and monitoring may indirectly benefit ER and PM [10,42,43].

#### 2.4.6. Caregiver Psychoeducation Component

To support generalization and caregiver mediation, the weekly psychoeducation for parents/teachers will follow established occupational therapy models. Specifically, Cog-Fun and POET typically deliver 6–10 weekly sessions and have demonstrated improvements in parental self-efficacy, adherence, and child functioning/participation, with effects maintained at follow-up in school-age samples [17,19,20,44]. Accordingly, the six-session parent/teacher component will be adopted to maximize transfer and contextualization of child strategies in daily routines. Given this dosing, three-month results should be interpreted with caution, and future research may evaluate protocols with a greater number of sessions or longer follow-up to determine the sustainability of effects.

#### 2.4.7. Session Structure and Rationale for Duration

Each session will last approximately 45 min, a duration selected to allow coverage of multiple cognitive tasks and metacognitive strategies within a single block, thereby supporting the repeated practice necessary for executive function training. This time frame is consistent with prior digital ADHD interventions [45,46] and comparable to traditional occupational therapy programs such as POET and Cog-Fun [16,19]. The session structure incorporates short activity segments and gamified elements with immediate feedback to maintain attention and motivation.

#### 2.4.8. Participant Support and Supervision

Throughout the intervention, participants will have the support of an assigned therapist from the research team, available to address questions or issues via the application’s internal chat or email (for children/adolescents and legal guardians). In addition, two supervisory videoconferences will be scheduled—Week 1 and Week 3—to answer questions and assess progress. If necessary, users or guardians may request additional videoconferences via chat or email, which will be scheduled according to the availability of the research team and families.

#### 2.4.9. Digital Capture and Usage Metrics

The platform will automatically record number of completed tasks, tasks initiated but not finished, tasks not initiated, duration of activities, average session duration, and active usage rate.

#### 2.4.10. Therapist Training

To ensure standardized delivery, all occupational therapists administering the intervention will complete a 20-h training program before implementation. This program covers theoretical foundations, intervention procedures, and the use of digital tools, including protocols for remote engagement and troubleshooting in virtual environments.

#### 2.4.11. Fidelity Assurance Procedures

Treatment fidelity will be maintained through a structured process that includes session checklists completed online by clinicians after each session to confirm adherence to prescribed components and session objectives; weekly remote supervision meetings with the principal investigator to provide feedback and address protocol-adherence issues; and ongoing digital support for technical or procedural queries. Fidelity will be monitored and reported as part of the feasibility outcomes (see Statistical Analysis for details on measurement, thresholds, and inter-rater verification).

#### 2.4.12. Rationale for Six-Week Duration

The selected six-week duration is consistent with recent digital ADHD interventions showing that multi-week, multi-session protocols (including four-week cycles) are feasible, achieve high adherence, and can yield preliminary functional improvements—for example, a 4-week digital therapeutic with high session compliance and attentional gains in 8–12-year-olds [45], and a 4-week RCT delivering 12 sessions (3/week; 30 min) with acceptable retention (~89%) and improvements in parent-rated symptoms and executive function [46]. At the same time, the field has not established a definitive “optimal dosage” for digital ADHD interventions; systematic reviews will be considered when interpreting dose–response heterogeneity [47,48].

### 2.5. Adverse Event Monitoring and Reporting

Although the intervention is considered low risk, the protocol includes systematic monitoring of adverse events to ensure participant safety. Low-risk events may include mild fatigue, frustration, emotional discomfort, headache, eye strain, or transient anxiety during tasks. These events will be documented in an adverse event log and managed by pausing the session, providing reassurance, and adjusting task demands as needed.

Serious adverse events (SAE) are defined as any event resulting in hospitalization, significant disability, life-threatening condition, or severe psychological reaction (e.g., acute anxiety crisis or suicidal ideation). Although SAE are highly unlikely in this context, the protocol specifies that any such event will trigger immediate suspension of the intervention, provision of appropriate support, and notification of the principal investigator and the institutional ethics committee within 24 h.

Participants and caregivers will receive written and verbal instructions on how to report any adverse events during or after sessions. The research team will maintain a standardized log documenting the nature, severity, and resolution of each event. The absence or presence of adverse events will be summarized in the final feasibility report.

### 2.6. Timelines and Schedule

After participant selection, an initial in-person session will be held with the participants and their legal guardians. During this session, instructions for starting the study, information on how to use the DiverAcción platform, available communication channels, and data security measures will be provided. Initial data collection (baseline, T0) will also take place during this session.

Training sessions will be conducted over the following six weeks, entirely online and under the remote supervision of the research team’s therapists. Once the twelve sessions are completed, a second in-person data collection session (post-intervention, T1) will be held during the same week the intervention ends.

Finally, a third evaluation (follow-up, T2) will be conducted three months after the intervention ends to assess the maintenance of the effects and long-term adherence. The three-month follow-up interval was chosen based on prior research on executive function interventions in children and adolescents with ADHD, where short- to medium-term maintenance of effects is commonly assessed within a 3–6-month window. This timeframe is considered sufficient to detect initial trends in maintenance or potential decay of intervention benefits while balancing feasibility and participant burden in a feasibility study. Previous studies have reported sustained improvements in working memory, attention, and processing speed at three-month follow-up after cognitive or combined interventions [39,49,50]. Therefore, the three-month interval aligns with established practices and provides meaningful preliminary data on the durability of intervention effects.

Therefore, data collection will take place at three points in time:

- Baseline (T0): before the start of the intervention.
- Post-intervention (T1): immediately after the twelve sessions.
- Follow-up (T2): three months later.

All in-person sessions will be held in a classroom at the local University. The flowchart in Figure 1 shows the timeline of the different phases of the study.

### 2.7. Sample Size

The planned sample size is 30 children/adolescents. This number is considered adequate for feasibility and usability studies in children and adolescents [25,51], ensuring sufficient variability to identify barriers and facilitators without requiring a formal calculation of statistical power [52]. This study was designed as a pilot trial aimed at assessing the feasibility and acceptability of the intervention. The sample size (n = 30) is considered appropriate for feasibility objectives, as it allows for the evaluation of implementation aspects, adherence, usability, acceptability and participant satisfaction. However, this sample size is insufficient to establish the efficacy of the intervention; Therefore, the findings should be interpreted as preliminary and intended to inform the design of future randomized controlled trials with larger samples.

### 2.8. Variables and Measures

#### 2.8.1. Main Dependent Variables

- Feasibility will be assessed using automated metrics recorded by the platform, including the number of tasks completed, cognitive errors (omissions, commissions, impulsivity, sequencing, and monitoring), average session duration, and active usage rate. These indicators will be calculated based on the frequency of weekly participation [43,53]. Acceptability of the DiverAcción platform will be assessed using an adapted version of the Technology Acceptance Model (TAM). The questionnaire consists of 20 items grouped into six dimensions: Perceived ease of use (F), Perceived fun (DP), Intention to use (IU), Compatibility (C), Self-efficacy (AE) and Attitude towards use (AC). Each item is rated on a 5-point Likert scale (1 = strongly disagree, 5 = strongly agree), allowing for quantitative evaluation of user perceptions regarding usability, enjoyment, behavioral intention, and integration into daily routines. This adaptation ensures ecological validity by incorporating motivational and contextual factors relevant to telerehabilitation for ADHD. The TAM framework has been widely validated for predicting technology adoption and is considered a robust measure of acceptability in digital health interventions [54].
- Usability: Usability was evaluated using the System Usability The System Usability Scale (SUS) is a widely used standardized questionnaire for measuring the overall perception of the ease of use of systems and applications [55]. The SUS consists of 10 items that alternate between positive and negative statements, scored on a 5-point Likert scale (1 = “strongly disagree” to 5 = “strongly agree”). Responses are converted into a total score ranging from 0 to 100, where scores of 68 or higher are considered indicative of good usability. This instrument has demonstrated high reliability (α > 0.90) and validity in various contexts, making it a robust tool for evaluating user experience in digital environments.

#### 2.8.2. Secondary Dependent Variables

- User-friendly design. The perception of the system’s ease of use and appeal will be assessed using a visual analog scale from 1 to 10, where 1 indicates an unfriendly experience and 10 reflects a highly intuitive and appealing design. This measure will allow for a quick and comprehensive evaluation of the perceived usability by participants, complementing other user experience indicators. The simplicity of this scale will facilitate its application in digital environments and its interpretation in terms of satisfaction and accessibility [54].
- Participant satisfaction. Satisfaction will be assessed using the Participant Satisfaction Questionnaire, consisting of 8 items in 4-point Likert format (1 = not at all satisfied; 4 = very satisfied), with a total score ranging from 8 to 32. Higher scores will reflect a greater level of satisfaction with the intervention.
- Therapeutic Alliance: The therapeutic alliance will be assessed using the Working Alliance Inventory for Internet Interventions (WAI-I) [27]. The WAI-I consists of 12 items rated on a 5-point Likert scale (1 = “never”, 5 = “always”), yielding a total score ranging from 12 to 60, with higher scores indicating a stronger alliance. The instrument evaluates three core components of the alliance—bond, goals, and tasks—and has demonstrated strong psychometric properties in digital health contexts, supporting its reliability for assessing collaborative engagement in online interventions.
- Therapeutic attitudes of professionals towards technology will be assessed using the e-Therapist Attitudes Toward Technology (e-TAP-T) [56]. This questionnaire will consist of 12 items using a 7-point Likert scale (1 = strongly disagree; 7 = strongly agree), designed to explore the professional’s level of commitment and willingness to use technological tools in clinical practice. The total score will range from 12 to 84, with higher scores indicating greater acceptance and commitment to technology. The e-TAP-T is considered a reliable measure for understanding attitudes that can influence the effective implementation of digital interventions.

#### 2.8.3. Effects Evaluation (Pre-Post)

The impact assessment of DiverAcción will be conducted using a multimodal approach that integrates different perspectives. Information will be collected focusing on children and adolescents, evaluating changes in planning, organizational, time management, and emotional self-regulation skills through standardized tests and objective metrics recorded by the platform. In parallel, questionnaires will be administered to parents and teachers to identify improvements in daily performance, and the transfer of strategies to natural contexts, as well as parental self-efficacy and stress. The opinions of therapists will also be considered, providing data on clinical applicability, program adherence, and the quality of the digital interaction. This approach will allow for a comprehensive view of the intervention’s effect, combining quantitative and qualitative indicators to ensure a robust and contextualized interpretation (Table 2).

- Executive functioning will be assessed using the Behavior Rating Inventory of Executive Function, Second Edition (BRIEF-2) [57], in its parent and teacher forms. Both questionnaires employ a three-point Likert scale (Never, Sometimes, Often) to measure the frequency of behaviors associated with executive functioning in everyday contexts. The parent version will assess domains such as inhibition, emotional control, flexibility, working memory, planning and organization, and monitoring, while the teacher version will capture similar dimensions within the school setting. The combined application of the questionnaire will provide an ecological perspective, facilitating the identification of difficulties and the planning of tailored interventions. In addition, the adolescents themselves will complete a self-report on their executive functioning using the Teenage Executive Functioning Assessment (TEXI Inventory) [58]. The TEXI Inventory, comprised of self-report versions for adolescents and other-report versions for parents and teachers, aims to assess everyday executive functions such as working memory and inhibitory control. The questionnaire consists of 20 items distributed across two factors, and its administration follows standardized criteria in school and home settings. The psychometric robustness of the TEXI is supported by high internal reliability (α ≥ 0.85), split-form reliability (≥0.81), and strong inter-rater reliability between adolescent and parent reports (r = 0.82).
- Time management will be assessed using the Time-S questionnaire [59]. This instrument consists of items designed to measure the perception, organization, and control of time in daily and academic activities. It will be administered using a Likert scale, allowing for an overall score that reflects the level of competence in time management. Higher scores indicate a greater ability to plan, prioritize, and allocate time efficiently. The Time-S is considered an innovative and reliable tool for analyzing this dimension in educational and clinical contexts.
- The Emotion Regulation Questionnaire for Children and Adolescents (ERQ-CA) [60] will be administered to participating adolescents to assess their emotion regulation strategies in everyday life. The questionnaire will be administered digitally, after obtaining informed consent, in an environment supervised by the research team. Each participant will respond to the 10 items on a 5-point Likert scale (1 = “never” to 5 = “always”), obtaining independent scores for the two dimensions: Cognitive Reappraisal and Expressive Suppression. ERQ-CA scores are calculated by summing the scores for each subscale. Cognitive Reappraisal reflects the tendency to reinterpret situations to reduce their emotional impact; high scores indicate frequent use of adaptive strategies associated with better emotion regulation and fewer internalizing symptoms. Conversely, Expressive Suppression assesses the inhibition of emotional expression; high scores are associated with less affective expression and greater psychological distress. A balanced profile is characterized by high reappraisal and low suppression, while low scores in both dimensions may suggest difficulties in emotional regulation.
- Prospective and retrospective memory will be assessed using the Prospective and Retrospective Memory Questionnaire for Children (PRMQ-C) [61]. This self-report instrument employs a five-point Likert scale (1 = never; 5 = very often) to measure the frequency of everyday memory lapses in natural contexts. The questionnaire includes items targeting both prospective memory (remembering to carry out intended actions) and retrospective memory (recalling past information), allowing for a differentiated analysis of these components. Psychometric studies report adequate internal consistency (Cronbach’s α > 0.80) and good construct validity, supporting its use in both clinical and educational settings. The ecological nature of the PRMQ-C facilitates the identification of memory difficulties in daily life and supports the design of tailored interventions to enhance functional memory performance.
- The KIDSCREEN-52 is a standardized, self-report questionnaire created to evaluate health-related quality of life (HRQoL) in children and adolescents aged 8–18. It includes 52 items across 10 dimensions: physical well-being, psychological well-being, moods & emotions, self-perception, autonomy, parent relations & family life, financial resources, social support & peers, school environment, and social acceptance (bullying). Built on a multidimensional framework, it captures a comprehensive view of physical, emotional, and social well-being. Psychometrically, it shows high internal consistency (Cronbach’s α = 0.77–0.89 per subscale) and satisfies test–retest reliability, convergent and discriminant validity, as evidenced in a representative European sample (n ≈ 22,800). The instrument takes approximately 15–20 min to complete and is suitable for epidemiological surveys, clinical research, and evaluation of interventions targeting pediatric populations.
- The Brief Parental Self-Efficacy Scale (BPSES) [62], assesses parents’ perceptions of their parenting competence. The instrument consists of a reduced, 5-item version, tailored to a single construct of parental self-efficacy. Each item is answered using a 5-point Likert scale, generating a total score ranging from 5 to 25. The BPSES has demonstrated satisfactory psychometric properties: factor analysis confirmed a unidimensional solution (CFI = 0.96, RMSEA = 0.094), and the 5-item version achieved adequate internal reliability (α = 0.75).
- The Parenting Stress Index, Fourth The Parental Stress Index (PSI-4-SF) [63,64] will be used to assess parental stress levels throughout the study. This instrument is a widely validated measure that identifies the degree of strain experienced by caregivers in relation to the demands of the parental role. The PSI-4-SF consists of 36 items distributed across three subscales: Parental Stress, Dysfunctional Parent-Child Interaction, and Child Difficulty, each designed to capture different dimensions of stress within family dynamics. Responses are recorded on a 5-point Likert scale (from “strongly disagree” to “strongly agree”), generating scores that reflect both overall stress and stress specific to each subscale. Previous studies have reported excellent internal consistency for the abbreviated version, with Cronbach’s alpha coefficients greater than 0.90 at different assessment points (α = 0.91, 0.91, and 0.92), supporting its reliability and temporal stability.
- Time Management Scale for Teachers (EMT) [65]. The EMT is a questionnaire designed for teachers to assess their students’ time management in everyday school situations. It consists of 23 items using a Likert scale from 0 (never/rarely) to 3 (always/almost always), where higher scores indicate greater mastery of the time dimension. It has a two-factor structure: time used for tasks and time used for events. Internal consistency reliability is excellent: α = 0.95 for the total scale, α = 0.94 for Factor 1, and α = 0.90 for Factor 2. Higher scores indicate better time management.

#### 2.8.4. Independent Variables

Sociodemographic, educational and economic data (age, gender, educational level, school year, residence) and preferences regarding digital intervention format (online, face-to-face or hybrid), previous experience with digital interventions and ADHD symptomatology and associated behavioral difficulties, through SNAP-IV [66]. This instrument consists of 26 items rated on a 4-point Likert scale (0 = not at all, 3 = very much), covering three dimensions: Inattention (9 items), Hyperactivity/Impulsivity (9 items), and Oppositional Defiant Disorder (8 items). Higher scores indicate greater symptom severity. The SNAP-IV has demonstrated strong psychometric properties, including high internal consistency and validity for clinical and research settings [66].

### 2.9. Statistical Analysis

The statistical analysis plan was developed to reflect the primary aim of this feasibility study: to assess processes and practicality rather than confirm efficacy. Therefore, analyses will emphasize descriptive statistics and estimation of precision for feasibility metrics (e.g., recruitment, retention, adherence, fidelity, and data completeness), as recommended by CONSORT guidelines for pilot and feasibility trials [52,67]. Adherence thresholds (≥80% for agenda use and training, and 100% for videoconferences) were selected to ensure meaningful engagement with the online intervention. Fidelity procedures, including a 20-h therapist training course and structured supervision, aim to guarantee consistent delivery of the protocol. Missing data will be primarily described, with multiple imputation reserved for sensitivity analyses of exploratory outcomes, reflecting best practices for small-sample feasibility designs.

#### 2.9.1. Outcome Assessment and Bias Minimization

Outcome assessors will follow standardized administration and scoring procedures and will be blinded to intervention delivery, with no access to fidelity forms, session plans, or platform usage data. Self-report and performance-based measures will be administered through secure digital tools, with automated time stamps.

#### 2.9.2. Feasibility Outcomes

**Figure 2:**
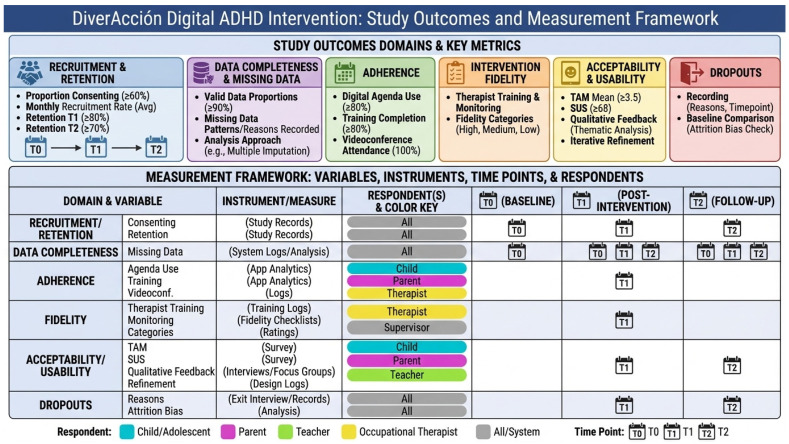
Feasibility Assessment of DiverAcción Digital ADHD Intervention: Measurement Model and Key Metrics.

The primary aim of this study is to assess feasibility across multiple domains, following established frameworks for pilot and feasibility trials [52,67,68]. Progression criteria for feasibility outcomes are summarized in Table 3 and Figure 2.

The following outcomes will be evaluated:

*Recruitment and Retention*

Recruitment will be measured as the proportion of eligible participants who consent to participate, and the average recruitment rate per month. Retention will be defined as the percentage of participants who complete all planned assessments at T1 (post-intervention) and T2 (follow-up).

*Data Completeness and Missing Data*

Data completeness will be calculated as the proportion of participants providing valid data for each measure at each time point. Patterns and reasons for missing data will be documented. This information will inform the feasibility of data collection procedures and guide refinements for a future trial. Primary feasibility analyses will use complete cases. Sensitivity analyses using multiple imputation may be performed for exploratory clinical outcomes, but the results will be interpreted with caution given the small sample size.

*Adherence*

Adherence will be assessed based on three components:

(a) use of the digital agenda on at least 80% of intervention days (over 6 weeks),
(b) completion of at least 80% of scheduled online training activities, and
(c) attendance at 100% of planned videoconference sessions.

Adherence will be reported as the proportion of participants meeting these criteria.

*Intervention Fidelity*

Fidelity will be ensured through a structured process: all occupational therapists delivering the intervention will complete a 20-h training course prior to implementation, covering theoretical foundations, intervention procedures, and use of digital tools. Fidelity will be monitored using session checklists, regular supervision meetings, and ongoing remote support. Fidelity will be categorized as high (≥80% of protocol elements delivered), moderate (60–79%), or low (<60%).

*Acceptability and Usability*

Acceptability will be explored through participant feedback collected via post-intervention questionnaires (TAM) [71], with a predefined threshold of mean ≥ 3.5 [70] and qualitative comments during videoconferences. Usability will be assessed using the System Usability Scale (SUS), considering scores ≥ 68 as acceptable [55]. Indicators will include perceived usefulness, ease of use, and satisfaction with the intervention format.

In the usability and acceptability evaluations, participants will have the opportunity to provide open-ended comments about their experience. These qualitative data will be recorded and analyzed using Atlas.ti 25 (25.0.2) software, following an inductive thematic analysis approach. Findings will inform iterative refinement of the intervention interface, task structure, and support strategies.

*Dropouts*

**Figure 3:**
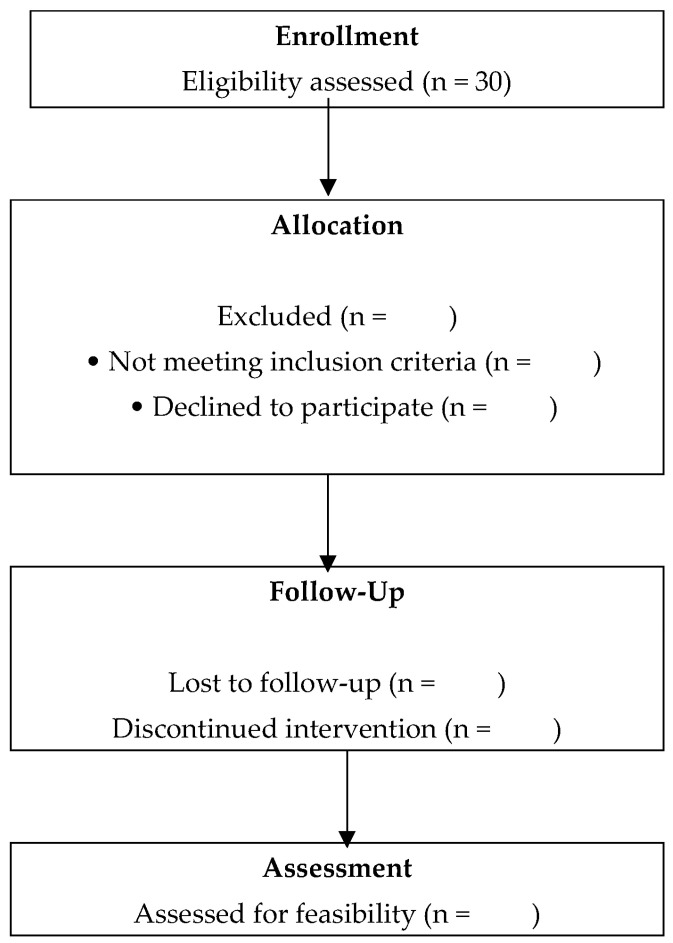
CONSORT Flow Diagram Adapted for Feasibility Trials. It will be completed after the collection.

Dropouts will be recorded with reasons when available. Descriptive analyses will compare baseline characteristics (e.g., age, sex, cognitive scores) between completers and non-completers to assess potential attrition bias. Participant flow will be presented using a CONSORT-style diagram adapted for feasibility trials (Figure 3).

#### 2.9.3. Exploratory Clinical Analyses

Exploratory analyses of clinical outcomes will aim to estimate variability and preliminary trends, not to draw confirmatory conclusions. Where appropriate, repeated-measures ANOVA or the nonparametric Friedman test will be used to compare results at different time points, prioritizing effect sizes and confidence intervals over *p*-values. Bonferroni-corrected post-hoc analyses will be performed for exploratory purposes only. Statistical significance will be set at *p* < 0.05 for these tests, but interpretation will be cautious. All analyses will be performed using IBM SPSS Statistics v28.

#### 2.9.4. Contextual Variables: Concomitant Care, Medication, and External Events

Concurrent services (type, frequency, dosage) will be documented at baseline and weekly; ADHD medication regimen (drug, dose, schedule) and any changes will be recorded weekly for applicable participants. School calendar events and major life changes will be logged to contextualize adherence and outcomes. Analyses will include sensitivity checks stratifying by concomitant care intensity and medication stability (stable vs. changed).

### 2.10. Data Management

Data will be stored securely and with restricted access, in accordance with national and European legislation on personal data protection and the institutional guidelines of the local University. The identity of participants will be protected in accordance with Organic Law 3/2018, of December 5, on the Protection of Personal Data and Guarantee of Digital Rights, and with Regulation (EU) 2016/679, the General Data Protection Regulation (GDPR).

Since the participants are minors, written informed consent will be obtained from their legal guardians, along with signed assent from the minor, after receiving a detailed explanation of the study’s objectives and procedures.

Each participant will be identified by a unique numerical code, used to pseudonymize all collected information. The table of correspondence between codes and identities will be stored in an encrypted file, separate from the consent forms and data files.

Data will be obtained through standardized assessments and automatic logs generated by the DiverAcción website (e.g., number of tasks completed, errors, execution times, or usage metrics). Digital files will be reviewed periodically to verify their integrity and quality before statistical analysis. All digital information will be stored on the University’s institutional cloud storage service, which complies with security standards and guidelines on personal data protection.

Access to the data will be restricted exclusively to authorized researchers, using multi-factor authentication and secure passwords. Physical documents (informed consent forms and administrative records) will be kept in locked filing cabinets on University premises.

Once the study is completed, personally identifiable data will be deleted. Only fully pseudonymized data may be shared for scientific purposes or deposited in public repositories, with the explicit prior consent of the legal guardians.

Legal guardians and participants may exercise their rights of access, rectification, erasure, or objection at any time by contacting the principal investigator as indicated in the study information sheet.

### 2.11. Patient and Public Participation

The development of the DiverAcción telerehabilitation system was carried out using a participatory approach, incorporating the perspectives of end users—adolescents with ADHD, their families, and educational and clinical professionals—from the initial phases of the project.

During the design phase, focus groups were conducted with adolescents, family members, and specialists to identify functional needs, format preferences, digital accessibility barriers, and motivational strategies in therapeutic contexts. These contributions helped define the system’s priority content and functionalities and ensure its relevance and practical applicability. Families also collaborated in reviewing the informational and consent materials, ensuring that the language used was clear, understandable, and appropriate for minors.

In this phase of the feasibility study, participants will not be involved in the methodological design or the analysis of the results, but they will receive regular updates on the project’s progress and its main findings. The study results will be presented to family associations and collaborating entities (educational centers and healthcare services) to facilitate community feedback and guide future steps toward a larger-scale evaluation.

### 2.12. Ethics and Dissemination

The DiverAcción study will be conducted in accordance with the ethical principles of the Declaration of Helsinki and applicable national and international regulations for research involving human subjects. Respect for the autonomy, privacy, and protection of participating minors and their families will be always guaranteed.

The protocol has been approved by the Local Biomedical Research Ethics Committee and complies with the requirements of Royal Decree 192/2023, of 21 March, which regulates medical devices, and Royal Decree 1090/2015, of 4 December, concerning clinical trials with medicinal products.

Since the participants are minors, their inclusion in the study will require the written informed consent of their legal guardians and the minor’s assent, after they have received clear information adapted to their level of understanding about the objectives, procedures, potential risks, and benefits of the study. Participation will be voluntary and may be discontinued at any time without negative consequences.

The DiverAcción telerehabilitation web platform will be provided free of charge by the local University, which will maintain the technical records and version control of the system used for research purposes. A member of the research team will be responsible for the supervision and proper use of the system during the study.

The personal and clinical data of the participants will be processed in a pseudonymous manner and in accordance with current legislation, as detailed in the data management plan included in the Data Management section.

The results of the study will be disseminated through publications in peer-reviewed scientific journals, national and international conferences, and institutional channels of the University of Granada. In addition, informative summaries will be prepared for participating families and associations, with the aim of facilitating the understanding and application of the findings in clinical and educational contexts.

## 3. Discussion

This study aims to examine the feasibility, usability, acceptability, and preliminary functional effects of DiverAcción, a telerehabilitation system designed to enhance planning, organization, time management, and emotional self-regulation skills in children and adolescents with Attention-Deficit/Hyperactivity Disorder (ADHD). The present protocol is part of an incremental development strategy aligned with international recommendations for the evaluation of complex interventions [29,30]. Consistent with our working hypotheses, establishing methodological feasibility and user acceptability is an essential prerequisite before progressing to a randomized controlled trial. A key strength of the intervention lies in its innovative focus on functional cognition within ecologically valid digital environments. Prior research has highlighted the limitations of traditional cognitive training, particularly its weak generalization to real-world contexts. In contrast, DiverAcción integrates gamified, interactive tasks designed to support the transfer of executive strategies to everyday situations, thereby fostering motivation and engagement—factors strongly associated with improved adherence in digital mental health interventions.

A key feature of DiverAcción is its collaborative calendar, designed for daily use in multiple contexts (school, leisure, self-care, family activities). This component not only facilitates planning and time management but also provides an integrated mechanism for promoting transfer to everyday life. Participants categorize each scheduled activity (e.g., school, self-care, leisure, sports), indicate its priority, estimate its duration, and rate its completion, generating objective data on engagement and participation in natural settings. These functionalities were prioritized during the co-design phase with families, participants, therapists, and teachers to ensure their validity and ecological relevance.

The collaborative agenda is conceptually aligned with Toglia’s multicontext approach, which emphasizes generalization through varied environments, explicit transfer criteria, metacognitive training, strategy-focused processing, and engagement in meaningful activities, the collaborative agenda operationalizes guided learning and self-control in multiple everyday contexts to strengthen functional [14,72,73]. In comparison with established occupational therapy programs, such as Cog-Fun (children) and Teen Cog-Fun (adolescents), which offer parent-child metacognitive strategy training in structured weekly sessions and typically assess outcomes using informant-reported measures, such as the Canadian Measure of Occupational Performance (COPM) and the Behavioral Rating Inventory of Executive Function (BRIEF), DiverAcción’s collaborative agenda adds ecologically valid in situ activity traces (e.g., prioritizing, estimating duration, completion) that can complement ratings and support monitoring of real-world transfer [16,17,44,74,75].

Similarly, the parent-centered POET program for preschoolers, delivered in eight sessions, has shown short-term improvements in executive functions and daily routine management, although its assessment relies primarily on parent-reported questionnaires and goal achievement rather than continuous digital recording in naturalistic settings. Therefore, the DiverAcción approach can complement the results of the POET-style approach by capturing objective signals of engagement in different environments [19,21]. In addition to these features, DiverAcción incorporates two scheduled videoconferences, one after the first week and another in the third week, to provide structured feedback and support. If families, teachers, or participants require additional sessions, these can be requested on demand. Furthermore, the platform includes a training section to improve various executive functions, emotional self-regulation, and prospective memory, as well as an integrated communication tool, similar to WhatsApp-style chat, that allows for continuous and direct interaction between participants and professionals throughout the intervention. While these indicators suggest potential transfer beyond the training context, generalization is not directly tested through experimental measures. This limitation should be considered when interpreting the findings, and future studies should include explicit assessments of functional generalization.

In contrast to the assumption that family involvement in DiverAcción is primarily psychoeducational, the protocol incorporates active family mediation and routine-based practice throughout the intervention period. Parents are required to support regular use of the platform and the collaborative agenda, establish consistent daily routines (e.g., homework, personal care, organization of materials), and apply executive and self-regulation strategies in the home context. These aspects are explicitly addressed during scheduled videoconference sessions with parents, where goal setting, supervision strategies, environmental structuring, and family stress management are discussed and adapted as needed. Importantly, family-mediated change is not only encouraged but also monitored through a multi-informant assessment strategy, including parent-, teacher-, and self-reported measures of executive functioning, emotional regulation, time management, parental stress, and perceived parental competence. This approach allows indirect monitoring of family-level and contextual changes over time.

Nevertheless, DiverAcción differs from highly manualized programs such as Cog-Fun and POET in that family-mediated practice is embedded within a flexible, digitally supported framework rather than prescribed through standardized homework protocols with formal fidelity checklists. This distinction should be considered when interpreting generalization processes and highlights an area for further refinement in future controlled trials.

A notable difference from Teen Cog-Fun is the intensity of family involvement. Whereas Teen Cog-Fun includes only two sessions for parents, DiverAcción provides six dedicated sessions (within an eight-session structure) for parents and teachers. It is reasonable to assume that two sessions may not be sufficient to address the substantial parental challenges of raising an adolescent with ADHD, as suggested by Theule et al. [76], who reported lower satisfaction regarding parents’ own achievements in parental self-efficacy. This expanded involvement in DiverAcción aims to strengthen parental competence and reduce stress through ongoing guidance and interactive support mechanisms.

The multidimensional assessment strategy employed—combining feasibility measures, usability, acceptability, satisfaction, and functional outcomes—aligns with prior feasibility studies and strengthens the validity of the evaluation. Automated metrics captured by the platform reduce the risk of observer bias and provide objective indicators of engagement, as recommended in recent digital intervention research. Furthermore, the active involvement of healthcare, educational, and family contexts supports the ecological applicability of the system and increases its potential for real-world implementation. The participatory development process, which incorporated perspectives from adolescents, families, and professionals, also reflects current best practices in user-centered digital health design. However, these aspects should be interpreted as preliminary and descriptive rather than confirmatory, given the exploratory nature of the study.

Several limitations must be acknowledged. First, as a pilot feasibility study, the absence of a control group and the modest sample size limit the ability to draw conclusions about efficacy. Second, external factors inherent to digital interventions—such as variability in access to devices, internet connectivity, and differences in technological proficiency—may influence user performance and engagement. Third, the wide age range (9–17) entails nonlinear changes in executive functioning and emotion regulation-; time versus -eventbased prospective memory places different demands on monitoring and cue use [3,34]. Although ageband delivery, scalable tasks, ER scaffolds, and interface adaptations were implemented, residual variability is likely; future trials should stratify by age and -prespecify PM subtype analyses (event vs. -timebased-) [9,11]. Likewise, SES and maternal education may moderate the development of executive functions, prospective memory, selfregulation, and participation in the intervention; these were not primary in this feasibility phase and justify stratification or a priori adjustment in confirmatory trials- [77,78]. Finally, because recruitment will be limited to a single region, caution is warranted regarding generalizability. Cultural, linguistic, and socioeconomic factors may influence engagement and outcomes; therefore, future trials are planned to incorporate multi-site recruitment, stratified sampling, and cross-cultural adaptation procedures to enhance external validity. These limitations highlight the need for caution when interpreting any observed trends and reinforce the importance of subsequent confirmatory trials. This protocol represents an initial step toward evaluating DiverAcción, focusing on feasibility and acceptability rather than efficacy. The findings will primarily inform improvements to the interface, task structure, and evaluation procedures, supporting the design of a larger randomized controlled trial. Future research should prospectively examine long-term outcomes, mechanisms of change, moderators of response (e.g., ADHD subtype, parental involvement), and implementation factors in clinical and educational settings. Collectively, this work aims to contribute to the development of accessible and ecologically valid digital interventions for adolescents with ADHD, pending further empirical validation.

## 4. Conclusions

This protocol establishes the foundation for evaluating the feasibility, usability, acceptability, and adherence of DiverAcción, a digital intervention designed to improve planning, organization, time management, prospective memory, and emotional self-regulation in young people aged 9 to 17 with ADHD. The combination of gamified tasks, a collaborative daily planner, and a structured psychoeducational program will allow for the collection of precise information on the system’s implementation in real-world contexts. The results are expected to provide preliminary evidence on satisfaction, ease of use, and the intention to continue using the program among children, adolescents, families, teachers, and professionals. Furthermore, the automated metrics will provide objective data that will facilitate the identification of barriers and opportunities for improvement. The findings from this study will guide the necessary adjustments to the platform and inform decisions regarding the feasibility of conducting a randomized clinical trial. In addition, the described methodology offers a replicable framework for future research on telerehabilitation and executive function training in children and adolescents. Taken together, this protocol represents an essential first step in optimizing DiverAcción and moving towards more accessible, effective, and environmentally valid digital interventions for young people with ADHD.

## Figures and Tables

**Table 1:** DiverAcción Program Structure by Target Group.

Dimension	Frequency	Main Aim	Description	Administered To
Collaborative Agenda	Daily (7 days/week)	Promote planning and monitoring in natural contexts	Participants co-design a personal agenda to schedule daily activities, prioritize tasks, estimate duration, and monitor completion.	Children/Adolescents
Training Sessions	12 sessions (2/week, 45 min each, 6 weeks)	Develop executive skills and emotional regulation through gamified tasks	Virtual sessions focused on planning, organization, time management, and emotional self-regulation using interactive exercises.	Children/Adolescents
Psychoeducation Sessions	6 sessions (1/week, 45 min each, 6 weeks)	Support generalization and caregiver mediation	Sessions for parents and teachers to reinforce strategies, manage routines, and reduce stress.	Parents/Teachers
Therapeutic Support	Continuous (chat/email) + 2 videoconferences (weeks 1 and 3)	Provide guidance and resolve doubts	Integrated communication tools and scheduled videoconferences to monitor progress and address questions.	Children/Adolescents & Parents/Teachers

**Table 2:** Variables, instruments and timing of administration.

Variables	Instruments	Time Data Collection	Administered to:
Previous experience with digital interventions, sociodemographic, educational and economic data of children and adolescents, pharmacological treatment, other behavioral or psychoeducational interventions	interview	T0	Parents
Preferences regarding digital intervention format	interview	T0	Children and Adolescents
Symptom severity in inattention, hyperactivity–impulsivity and oppositional behaviors (not for subtype classification)	SNAP-IV	T0	Parents
Therapeutic attitudes of professionals towards technology	e-TAP-T	T0	Teachers Occupational therapists
IQ estimated	WASI-II	T0	Children and Adolescents
Feasibility	Automated metrics recorded by the platform: adherence, number of uses, sessions, time of use, errors	T1	Children and Adolescents Parents Teacher Occupational Therapists
Acceptability	TAM questionnaire	T1	Children and Adolescents Parents Teacher Occupational Therapists
Usability	SUS	T1	Children and Adolescents Parents Teacher Occupational Therapists
User-friendly design	Visual analog scale from 1 to 10	T1	Children and Adolescents Parents Teacher Occupational Therapists
Participant satisfaction	Participant Satisfaction Questionnaire	T1	Children and Adolescents Parents Teacher Occupational Therapists
Therapeutic Alliance	WAI-I	T1	Children and Adolescents
Executive functioning	BRIEF-2 for parents and teachers TEXI Inventory	T0, T1, T2	Parents and Teachers Children and Adolescents
Time management of children and adolescents	Time-S questionnaire EMT	T0, T1, T2	Children and Adolescents Teachers
Prospective Memory	PRMQ-C Automated metrics recorded by the platform	T0, T1, T2 T1	Children and Adolescents
Emotion regulation strategies in everyday life	ERQ-CA	T0, T1, T2	Children and Adolescents
Health-related quality of life (HRQoL)	KIDSCREEN-52	T0, T1, T2	Children and Adolescents
Parents’ perceptions of their parenting competence	BPSES	T0, T1, T2	Parents
Parental stress levels	PSI-4-SF	T0, T1, T2	Parents

Note: T0 = baseline, T1 = immediately post-intervention, T2 = 3 months after intervention.

**Table 3:** Progression Criteria for Feasibility Outcomes.

Domain	GO (Proceed)	AMEND (Modify)	STOP (Do Not Proceed)
Recruitment	≥70% of eligible approached	50–59%	<50%
Retention	≥80% at T1/T2	60–79%	<60%
Data completeness	≥80% completeness	70–79%	<70%
Adherence	≥80% agenda & training; 100% videoconferences	60–79% agenda/training	<60% agenda/training
Fidelity	≥80% protocol elements delivered	60–79%	<60%
Acceptability (TAM)	Mean ≥ 3.5	Mean 3.0–3.49	Mean < 3.0
Usability (SUS)	Score ≥ 68	Score 60–67	Score < 60
Dropouts	≤20% dropout rate	21–29% dropout rate	≥30% dropout rate

Note. GO = criteria met for progression to definitive trial; AMEND = criteria met with modifications; STOP = criteria not met. Predefined success thresholds were based on widely used benchmarks in feasibility research, such as ≥80% session completion [69], SUS ≥ 68 [55], and TAM mean ≥ 3.5 [70].

## Data Availability

The data cannot be shared publicly due to privacy and ethical restrictions, in accordance with the guidelines of the Oficina de Protección de Datos de la Universidad de Granada (UGR). However, the data can be made available upon reasonable request to the corresponding author, if compliance with confidentiality and data protection regulations is ensured.

## Supplementary Materials

The following supporting information can be downloaded at: https://www.mdpi.com/article/10.3390/healthcare14030323/s1.
