# Supplementary material for: Feasibility of “DiverAcción”: A Web-Based Telerehabilitation System for Executive Functions Training in Children and Adolescents with ADHD—Longitudinal Study Protocol
Source: Healthcare (Basel). 2026 Jan 27;14(3):323. doi: 10.3390/healthcare14030323 (PMC12896990; doi:10.3390/healthcare14030323)
Supplement: Supplementary file 1 [file healthcare-14-00323-s001.zip › healthcare-4067839-supplementary.pdf]

Supplementary Material

Adaptation of the CONSORT Extension for Pilot and Feasibility Trials, SPIRIT, and CONSORT eHealth Reporting Checklist

Domain	Key Item	Compliant (Yes/No)	Notes / Action
Title and Abstract	Indicates that it is a pilot/feasibility and digital study	Yes	The title mentions Feasibility and Web-Based Telerehabilitation
Introduction	Justification of the study and specific feasibility objectives	Yes	The need and objectives are explained
Design	Type of study (pilot, randomized, etc.) and justification	Yes	Quasi-experimental, following MRC and SPIRIT guidelines
Participants	Inclusion/exclusion criteria and context	Yes	Detailed in Section 2.3
Digital Intervention	Detailed description (technology, platform, access, security, usability)	Yes	Section 2.4, Figure 1, and Table 1; Sections 2.9 and 2.11
Outcomes	Defines primary and secondary feasibility measures (including success criteria, usability, and acceptability)	Yes	Section 2.7, Figure 2, and Table 2
Sample Size	Justification based on feasibility objectives	Yes	Section 2.6 indicates n = 30 and provides justification
Flow Diagram	Adapted CONSORT for feasibility	Partial (Not started)	Figure 3
Procedures	Timeline, randomization (if applicable), blinding	Yes	Timeline included; clarification on blinding is pending
Ethical Aspects	Consent, data protection, risks	Yes	Sections 2.9 and 2.11
Analysis	Statistical methods and how they will inform the definitive trial	Yes	Section 2.8 describes analysis and criteria
Digital Implementation	Usage context, technical support, adherence, usage metrics, and fidelity	Yes	Detailed in intervention and outcomes; Sections 2.2, 2.4, 2.7, and 2.8
Limitations	Risks and mitigation strategies	Yes	Discussion section includes limitations
Registration and Protocol	Registration on platform (ClinicalTrials, OSF)	Partial	Registration will be completed after feasibility results, to

	and reference to SPIRIT		incorporate finalised protocol amendments. Once registered, the registration number will be included in updated protocol document
